# Case report: First evidence of impressive efficacy of modulated dose selpercatinib in a young Caucasian with ANK3-RET fusion-positive NSCLC

**DOI:** 10.3389/fonc.2024.1307458

**Published:** 2024-02-07

**Authors:** Elisa De Carlo, Elisa Bertoli, Monica Schiappacassi, Brigida Stanzione, Alessandro Del Conte, Roberto Doliana, Michele Spina, Alessandra Bearz

**Affiliations:** ^1^ Department of Medical Oncology, Centro di Riferimento Oncologico di Aviano (CRO), Istituto di Ricovero e Cura a Carattere Scientifico (IRCCS), Aviano, Italy; ^2^ Department of Medicine (DAME), University of Udine, Udine, Italy; ^3^ Molecular Oncology Unit, Oncologia Molecolare e dei Modelli Preclinici di Progressione Tumorale (OMMPPT) Department of Translational Research, Centro di Riferimento Oncologico di Aviano (CRO), IRCCS, Aviano, Italy

**Keywords:** NSCLC, *RET* fusion, *RET* inhibitors, next-generation sequencing, selpercatinib

## Abstract

Over the past decade, molecular characterization has led to change the management of advanced non-small cell lung cancer (NSCLC) harboring driver mutations. Rearranged during transfection (*RET*) gene fusions, occurring in 1% to 2% of NSCLC, have emerged as an oncogenic druggable target. Systemic targeted therapies with highly selective *RET* inhibitors (RETi), selpercatinib and pralsetinib, represent a recent clinical breakthrough. While the development of RETi has improved survival, with their increasing use, it is crucial to be aware of the risks of rare but serious adverse events (AEs). A particular challenge for clinicians in applying targeted therapies is not only diagnosing but also interpreting rare mutations. Herein, we report a case of a 43-year-old Caucasian advanced NSCLC patient diagnosed with a rare *RET* gene fusion, *ANK3::RET*, identified with Next Generation Sequencing (NGS). Selpercatinib has been initiated at the recommended initial dose after one incomplete chemotherapy cycle due to a severe infusion reaction, but it subsequently required a dose adjustment following grade 3 (G3) AEs. During treatment, we used a particular selpercatinib dosage (160 mg in the morning and 80 mg in the evening) with good tolerance and without compromising effectiveness. Our finding broadens the range of *RET* fusion types in not-Asian NSCLC. To the best of our knowledge, our case demonstrates, for the first time, a clinical and radiological response to frontline highly selective RETi selpercatinib, expanding the spectrum of potential oncogenic *RET* fusion partners in newly diagnosed NSCLC patients. Furthermore, to our knowledge, this is the first case describing a *RET* fusion-positive (*RET*+) NSCLC patient treated with a modified selpercatinib dosage outside the drug data sheet and demonstrating a safe and effective use.

## Introduction

We are recently faced with a progressive paradigm shift in the treatment of advanced NSCLC, matching a specific targeted therapy for oncogenic driver alterations subsets (1). Molecular profiling with next-generation sequencing (NGS), detecting actionable driver mutations, is recommended by International Guidelines in newly diagnosed advanced NSCLC and led to the development of personalized therapeutic decision-making, specifically in adenocarcinoma (2, 3). *RET* is a proto-oncogene, located in the pericentromeric region of chromosome 10q11.2 that encodes a tyrosine kinase receptor involved in the development of neural crest-derived cell lineages, kidney, and male germ cells (4, 5). The *RET* oncogene activation occurs mainly in two different ways: chromosomal rearrangement and somatic or germline mutations. The *RET* fusions usually involve a 3’ sequence of the *RET* gene, encoding the kinase domain, and a 5’ sequence of other chaperone genes. The properties of the newly formed fusion proteins depend on the specific partner gene and the site of the *RET* breakpoints. Genomic rearrangements of *RET* gene could result in either ligand-independent activation or aberrant *RET* expression (4, 5).

The *RET* oncogene aberrations have been identified in various tumors. *RET* mutations have been reported in medullary thyroid carcinoma, with an incidence between 43% to 71% of sporadic cases; while germline mutations are a pathognomonic hallmark of multiple endocrine neoplasia (MEN), correlated with a high risk of developing a medullary thyroid carcinoma (6, 7). On the other hand, *RET* rearrangements are most common among papillary thyroid carcinoma in around 20-40% of patients (8, 9). Genomic rearrangements of *RET* gene occur in approximately 1-2% of NSCLC, mainly in young, non-smokers or light-smokers, adenocarcinoma patients (10–15). In NSCLC, the most frequently observed partners are kinesin family member 5B gene (*KIF5B*)::*RET* (7 variants) and coiled-coil domain containing 6 (*CCDC6*)::*RET* (16, 17). Other rearrangements are with Nuclear receptor coactivator 4 (*NCOA4)*, Ephrin type-A receptor 5 (*EPHA5)*, Unconventional myosin-Va *(MYO5C)*, tripartite motif containing 33 (*TRIM33)*, CAP-GLY domain containing linker protein 1 *(CLIP)*, ELKS/RAB6-interacting/CAST family member 1 *(ERC1)*, Phosphatidylinositol binding clathrin-assembly protein *(PICALM)*, FERM Domain Containing 4A *(FRMD4A)*, and RUN and FYVE domain containing 2 (*RUFY2)* (18, 19).

As an inciting oncogenic event, *RET* fusions rarely co-occur with other activating mutations, although they might appear as a resistance mechanism during different tyrosine kinase inhibitors treatments for druggable alterations (20, 21).

Retrospectively, Pemetrexed-based chemotherapy demonstrated optimal responses in *RET*+ NSCLC (22). Furthermore, *RET*-rearranged adenocarcinomas usually have low Programmed death-ligand 1 (PD-L1) expression, not by chance response to immunotherapy is poor (23). In the past decade, different multitarget agents, such as cabozantinib, vandetanib, sorafenib and lenvatinib, have shown ancillary RETi activity, reflecting the lack of selectivity of these drugs (14, 24). In recent years, selective oral RETi, selpercatinib (LOXO-292) and pralsetinib (BLU-667), have been developed (25, 26). Two global phase 1/2 clinical trials, LIBRETTO-001 and ARROW (27–30), demonstrated the improved activity of these RETi in both platinum-treated and naïve *RET*+ NSCLC patients, leading to approvals by US Food and Drug Administration (FDA) and European Medicines Agency (EMA). Herein, we report the case of a Caucasian young woman presented with stage IV lung adenocarcinoma, harboring a rare *RET* rearrangement, demonstrating an impressive clinical and radiologic response to selpercatinib.

## Case report

A 43-year-old female patient was admitted to the Emergency Department with epigastric pain, nausea and fever, with a temperature up to 38°C, in November 2022. She had a history of light smoking for a few years twenty years before. Chest and abdomen computed tomography CT scan revealed left pleural effusion, peritoneal carcinomatosis, liver metastasis and enlarged celiac and inter-aorto-caval lymphadenopathies. Brain magnetic resonance imaging (MRI) was negative for brain lesions.

**Figure 1:**
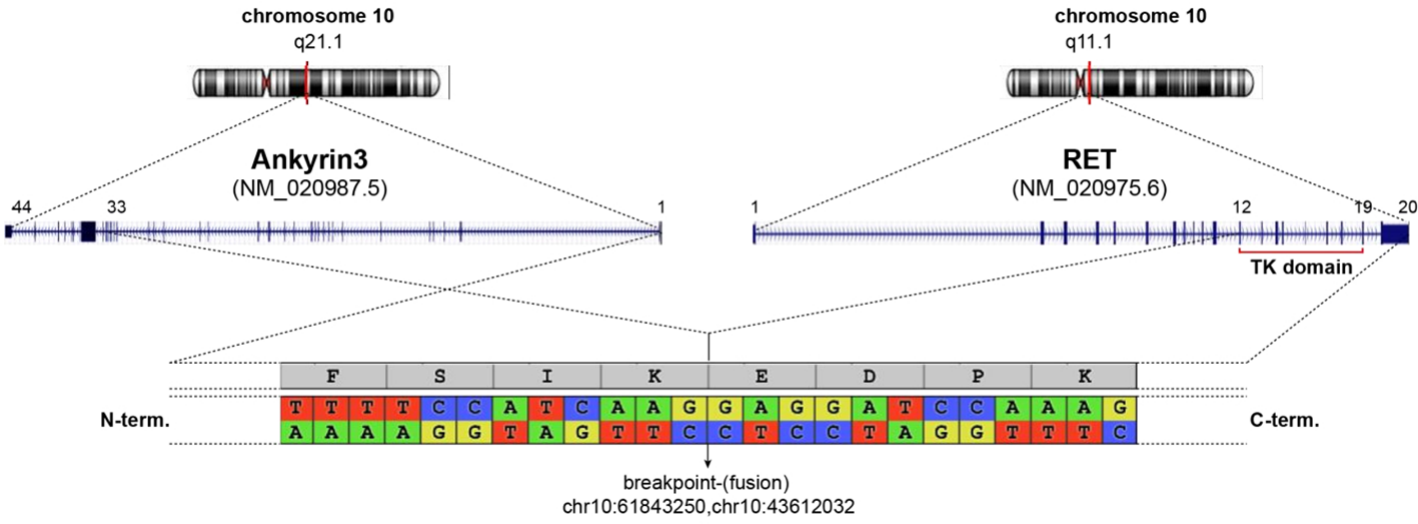
Schematic representation of ANK3-RET fusion detected. Both genes are located on chromosome 10 (upper graph). The detected fusion occurs as depicted in central graph linking ANK3 (exon 1-33) to RET (exon 12-20) given a chimeric protein (below). Breakpoint area is enlarge showing nucleotide sequence and coding aminoacids.

The patient underwent thoracentesis with 1200 ml of serum blood fluid drainage and talc pleurodesis. Cytological examination showed the presence of suspicious features for malignancy of epithelial cells. Subsequently, a diagnostic laparoscopy was performed. Histopathological examination revealed a pulmonary origin of peritoneal lesions. In particular, the results of immunohistochemistry (IHC) staining were positive for Cytokeratin7 (CK7), protein16 (p16), Anti-Thyroid Transcription Factor (TTF1), Napsin and negative for Paired box gene 8 (PAX8), Calretinin, GATA Binding Protein 3 (GATA3), Cytokeratin20 (CK20), Estrogen receptor (ER), Wilms’ tumor 1 (WT1), Caudal-type homeobox transcription factor 2 (CD-X2), protein53 (p53), allowing a definitive diagnosis of peritoneal localization of lung adenocarcinoma with acinar growth pattern; PD-L1 was <1%. Molecular profiling of the biopsy sample was carried on performing NGS. Gene fusion analysis on tumor Ribonucleic acid (RNA) revealed a rare Ankyrin3 (*ANK3*) (Exon 33)::*RET* (Exon 12) rearrangement (in 96% of the analyzed reads). The gene fusion detected generates a chimeric protein *ANK3-RET* composed of the amino-terminus of *ANK3* and the carboxyl-terminus of *RET*, including its kinase domain as depicted in **Figure 1**. Molecular alterations in driver genes, such as Epidermal growth factor receptor (*EGFR)*, Kirsten rat sarcoma virus *(KRAS)*, Erythroblastic oncogene B-2 *(ERBB2)*, Mesenchymal Epithelial Transition factor *(MET)* and V-Raf Murine Sarcoma Viral Oncogene Homolog B *(BRAF)* were not detected on tumor Deoxyribonucleic acid (DNA).

The patient was subsequently prescribed standard first-line treatment with carboplatin (AUC 5 i.v. every three weeks) and pemetrexed (500 mg/m² i.v. every three weeks). During first infusion of carboplatin, the patient reported a grade 3 infusion-related reaction after a few minutes; therefore, the chemotherapy infusion was interrupted (pemetrexed was completely administered while carboplatin only for few milligrams). Due to infusion reaction and to patient refusal, chemotherapy was definitively interrupted after the first cycle. In December 2022, because of worsening of dyspnoea needing oxygen therapy and uncontrolled abdominal pain, the patient started treatment with Selpercatinib at at the recommended initial dose of 160 mg twice a day, with immediate clinical benefit. On February 2023, a CT scan revealed a partial response (PR), with a reduction of pleural thickening, left pleural effusion and in the size of most omental nodules and liver lesions. The patient no longer required oxygen therapy.

In March 2023, the patient reported grade 3 (G3) fatigue, G2 chest, pelvic pain and dyspnoea and G3 hypertension. Electrocardiogram (ECG) revealed iatrogenic QT corrected (QTc) interval prolonged (516, G3); laboratory documented an Aspartate aminotransferase (AST), Alanine aminotransferase (ALT) and Gammaglutanyltransferase (GGT) G3 elevation. Other possible concomitant contributing factors for QTc prolongation were excluded: there were neither electrolyte disorders nor concomitant medications knowning to prolong QTc interval. The patient was hospitalized and Selpercatinib was temporarily discontinued. During hospitalization, a cardiological evaluation with ECG and echocardiography was performed, with evidence of non-specific inverted T waves, negative troponin and prescription of amlodipine 5 mg daily for hypertension. Moreover, abdominal MRI confirmed the stability of liver metastasis and did not show new pathological findings and other possible causes of hepatitis were excluded. After complete resolution of G3 hypertransaminasemia and QTc prolongation, returning to normal values, Selpercatinib was prescribed at the reduced dose of 80 mg twice a day. On April 2023, the CT scan displayed pleural effusion complete resolution with pleural thickening stability, liver lesions reduction mediastinal and abdominal lymphadenopathies disappearance and omental nodules stability.

**Figure 2:**
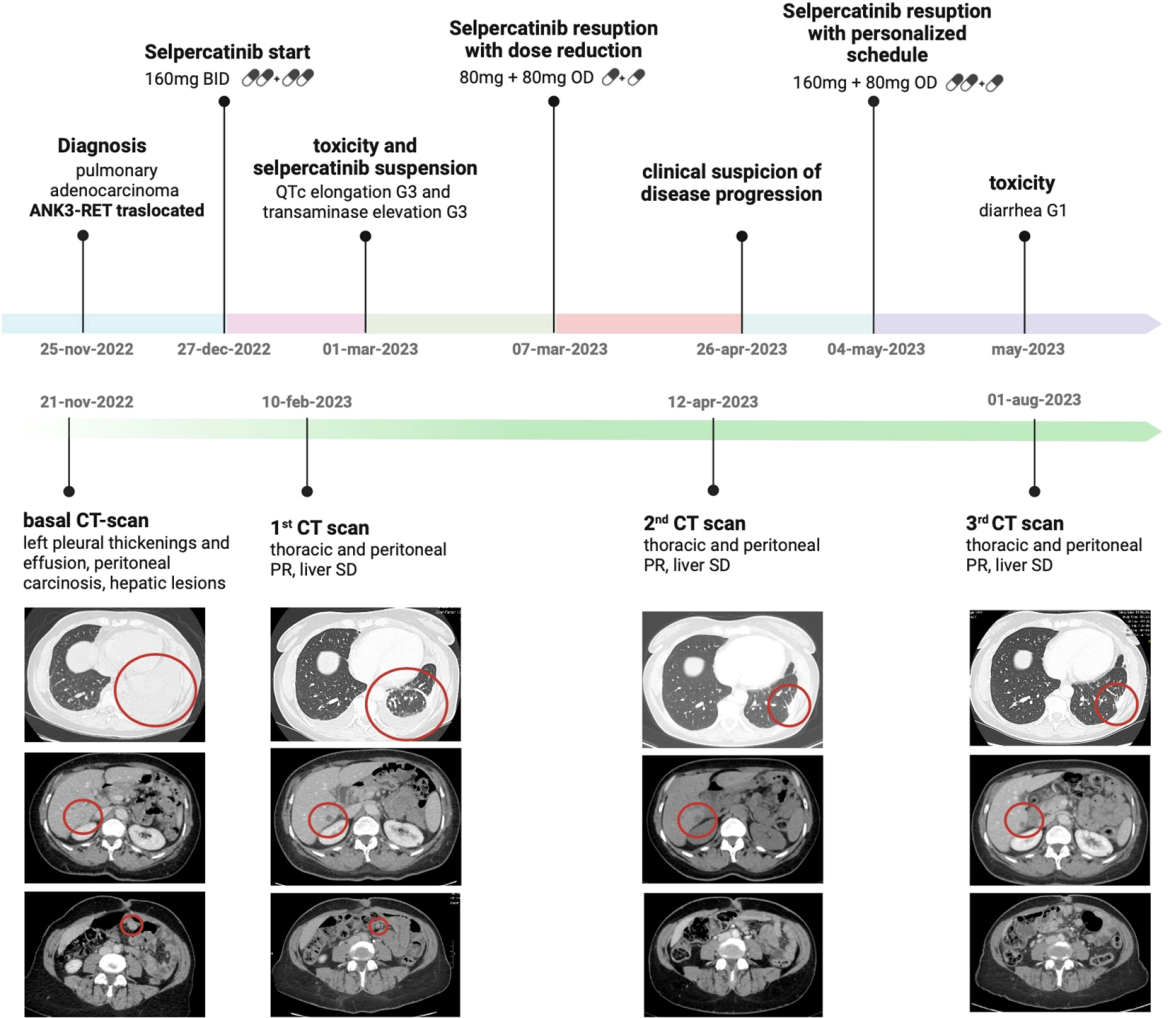
Patient’s clinical course.

At the end of April 2023 abdominal pain, present at the time of diagnosis, recurred intensively, without other clinical symptoms or blood tests alterations. Considering that symptoms occurred two weeks after the execution of the CT showing the maintenance of a partial overall disease response, given the difficulty of radiologically evaluating carcinosis and to avoid the exposition of the patient to a second extremely close contrast agent and ionizing radiation potentially needlessly, it was not considered clinically appropriate to repeat a CT.On May 2023, on the suspicion that the reduced dose drug did not effectively control the disease, Selpercatinib was prescribed at a dose of 160 mg in the morning and 80 mg in the evening. The reason for the choice of this particular dosage is related to the need to reach symptom control without inducing severe adverse events, particularly cardiological toxicity. This Selpercatinib dosage was moderately tolerated; patient reported diarrhoea G1, without cardiological adverse events, hypertension and without hypertransaminasemia; QTC intervals were normal at subsequent ECG controls. Abdominal pain was slightly reduced. On August 2023, CT scan revealed further partial response of pleural thickenings and liver metastases with unvaried peritoneal carcinosis; an encephalic MRI showed no evidence of brain disease. The patient is still under Selpercatinib treatment at 160mg plus 80mg daily dosage. **Figure 2** represents the patient’s clinical course.

## Methods

### Molecular profiling

Nucleic acids from the Formalin-Fixed Paraffin-Embedded (FFPE) biopsy tissue were extracted using QIAmpDNA FFPE extraction kit (Qiagen) and Maxwell 16 LEV RNA FFPE kit (Promega) according to the manufacturer’s protocols.

Molecular profiling was performed on extracted DNA using an *“in-house”* developed NGS panel covering multiple hot spots on *EGFR, ERBB2, MET, KRAS* and *BRAF* genes. Gene fusion analysis was performed on extracted RNA using Archer Fusion Plex Lung Cancer Kit which allows the detection of novel fusion partners. The library samples were run on an Illumina Miseq. Raw sequence data was analyzed using a pipeline based on Illumina Local Run manager and variant caller, Integrated Genome Viewer (IGV) and Varsome. For gene fusion analysis, fastq files were uploaded and analyzed on Archer Analysis 6.2 platform. Ref Sequences: *ANK3*: NM_020987.5, *RET* NM_020975.6).

## Discussion

*RET* fusion represents a new NSCLC subgroup with a targetable oncogenic driver. Many efforts have been made to target *RET* therapeutically over time; treatment for cancers harboring a *RET* fusion has evolved from traditional chemotherapy and non-selective multiple kinase inhibitors to selective RETi.

The new specific RETi, selpercatinib and pralsetinib have been registered by the US FDA and EMA on the basis of two multicenter, open-label, multicohort clinical trials. The activity of selpercatinib was demonstrated in the phase 1/2 LIBRETTO-001 trial (NCT03157128) in patients whose tumors had *RET* alterations (27). In this pivotal phase 1/2 clinical trial selpercatinib treatment was associated with remarkable responses in NSCLC patients both previously treated with platinum-based chemotherapy and treatment-naïve patients (28). The objective response rate (ORR) within *RET*+ NSCLC was 64% in patients treated with platinum-based chemotherapy and 85% in treatment-naive patients (28). In the phase 1/2 ARROW trial (NCT03037385), pralsetinib has been reported to be an effective treatment for *RET*+ NSCLC, demonstrating activity both in prior platinum-treated and treatment-naïve patients, with an ORR respectively of 61% and 70% (29, 30). It has been reported intracranial activity and a manageable safety profile, with mainly low-grade toxic effects, for both RETi (27–30).

Therefore, a precision treatment of *RET*+ NSCLC has been tailored.

In our case report, NGS was used to perform panel sequencing allowing the detection of a rare *RET* rearrangement, the *ANK3(33)*::*RET(12)* fusion. This rare rearrangement has been found only in 2 patients, with different fusions, in a large retrospective study of 1162 Chinese NSCLC patients (31). A case report has described *ANK3::RET* fusion as a druggable mechanism of osimertinib resistance; pralsetinib has demonstrated potent clinical activity when combined with osimertinib in treating an *EGFR*-mutated NSCLC Caucasian patient with *RET*-mediated mutation resistance (32). Another case report presents a case of *RET* dual fusion, *ANK3::RET* and *CCDC6::RET*, in an advanced Chinese NSCLC patient treated with pralsetinib after the failure of other treatments, including platinum-based chemotherapy (33).

Currently there is a limited understanding in the genomic landscape of NSCLC and other solid tumors harboring *RET* fusions. A retrospective multi-center study has recently investigated clinic-biological features and treatment outcomes of patients with any-stage *RET*+ NSCLC from 31 centers, 30 European and one from Argentina. The most frequent fusion partner was KIF5B followed by CCDC6; no cases were detected with *ANK3::RET* fusion (34). *RET* translocations/fusion genes are predominantly reported in papillary thyroid carcinoma and NSCLC, less in other solid tumors, such as colorectal carcinoma, salivary gland carcinoma, serous ovarian carcinoma, pancreatic and breast carcinoma, in global multicenter networks and reviews; *ANK3::RET* fusion was seen only in a papillary thyroid cancer (12, 17, 34, 35). The study of Parimi et al, reported the clinicopathologic and genomic landscape of a large cohort of *RET* fusion positive solid tumors (523 patients with NSCLC and 368 with other solid tumors), including the discovery of 61 novel fusions, detected by a DNA tissue-based NGS assay; no case of *ANK3::RET* rearrangement was detected in all African, Central and South American, East Asian, and South Asian patients (36).

The sensibility to highly selective RETi, selpercatinib and pralsetinib, was not demonstrated in naïve treatment lung adenocarcinoma with *ANK3::RET* rearrangement as unique oncogenic druggable target. In the Chinese study, the two *ANK3::RET*+ NSCLC patients did not receive targeted therapies (31) and in the two case reports pralsetinib was not administered as frontline treatment (32, 33).

To the best of our knowledge, this was the first case reported a novel rare *RET* fusion, *ANK(33)::RET(12)*, in a not-Asian newly diagnosed NSCLC patient; furthermore, because of in our case chemotherapy was incomplete administered for only one cycle due to severe infusion reaction, we could conclude that this is the first report describing clinical and radiological response to highly selective RETi in naïve treatment *ANK3::RET* positive NSCLC.

In the current era of precision medicine, this case exemplifies how comprehensive genomic profiling with NGS displays greater strengths in identifying fusion variants and may provide important treatment options also for rare molecular alterations.

In addition, this case highlights the unique challenge of using a modified schedule of selpercatinib (160 mg in the morning and 80 mg in the evening) not included in the drug data sheet, reporting patient benefit with low-grade side effects.

Selpercatinib is a first-in-class, highly selective, and potent intracranial RET kinase inhibitor. In the phase I-II LIBRETTO-001 trial selpercatinib was orally administered in a continuous 28-day cycle. Patients enrolled in the phase 1 dose-escalation portion received between 20 mg once daily or 20–240 mg twice daily; the phase 2 recommended dose was 160 mg twice daily (27, 28, 37). Selpercatinib had a manageable tolerability profile and was associated with mainly low-grade toxic effects; this finding is consistent with its RET-selective profile and lack of substantial off-target activity. Adverse events could generally be managed with dose reductions and only a small proportion of patients discontinued selpercatinib because of drug-related adverse events. The most common treatment-related AEs that were grade 3-4 in severity were hypertension, elevated alanine aminotransferase and elevated aspartate aminotransferase. The majority of grade 3 AEs were reversible with dose modifications; this finding suggests that long-term treatment is feasible, particularly in light of the responses observed with selpercatinib at doses as low as 20 mg once daily (27, 28, 37). Based on this case of treating a rare *RET*-fusion NSCLC patient, we retain that this novel rare fusion might be a potential oncogenic driver, sensitive to frontline selective RETi as selpercatinib. Furthermore, the peculiar dosage of selpercatinib might be safe and efficacious, although a careful risk-benefit discussion with the patient is needed. Despite its limited generalizability, our case reported a novel molecular target in Caucasian *RET* fusion NSCLC patients and demonstrated the efficacy of a modified and tailored selpercatinib dosage.

## Conclusion

Our finding broadens the range of *RET* fusion types in not-Asian NSCLC population and provide the basis for the hypothesis that highly selective RETi, such as selpercatinib, represent the best treatment option in naïve treatment *ANK3-RET* positive lung adenocarcinoma. Selpercatinib dose adjustment, outside the drug data sheet, reduced the frequency and intensity of AEs without compromising effectiveness, highlighting the benefit of tailoring doses to optimize treatment outcomes and supporting clinical decision-making.

## Data availability statement

The datasets for this article are not publicly available due to concerns regarding participant/patient anonymity. Requests to access the datasets should be directed to the corresponding author.

## Ethics statement

Written informed consent was obtained from the individual(s) for the publication of any potentially identifiable images or data included in this article.

## Author contributions

ED: Conceptualization, Data curation, Writing – original draft, Writing – review & editing, Validation. EB: Data curation, Supervision, Validation, Writing – review & editing. MSc: Methodology, Supervision, Validation, Writing – review & editing. BS: Data curation, Validation, Writing – review & editing. AD: Validation, Visualization, Writing – review & editing. RD: Methodology, Validation, Writing – review & editing. MSe: Supervision, Validation, Writing – review & editing. AB: Supervision, Validation, Writing – review & editing.
